# Micrometastasis of endometriosis to distant organs in a murine model

**DOI:** 10.18632/oncotarget.16889

**Published:** 2017-04-06

**Authors:** Elham N. Samani, Ramanaiah Mamillapalli, Fei Li, Levent Mutlu, Demetra Hufnagel, Graciela Krikun, Hugh S. Taylor

**Affiliations:** ^1^ Department of Obstetrics, Gynecology and Reproductive Sciences, Yale School of Medicine, Connecticut 06510, New Haven, USA

**Keywords:** endometriosis, micrometastasis, DsRed cells, metastasis, mice

## Abstract

Endometriosis is an inflammatory gynecological disorder among reproductive-aged women caused by the engraftment and proliferation of endometrial cells outside the uterus, most commonly in the pelvis. It is thought that the disease arises primarily from retrograde menstruation where cells from the endometrium travel through the fallopian tubes to the peritoneal cavity. However, migration of endometriosis-derived cells to distant organs outside of the peritoneal cavity have not been explored. In the present study, we developed and validated a mouse model of disseminated endometriosis using syngeneic DsRed endometrial tissue introduced into the peritoneum of immunocompetent mice. Flow cytometry and immunofluorescence analysis, demonstrated the presence of endometriosis-derived cells in multiple organs (including lung, spleen, liver and brain) in the murine endometriosis model. Immunostaining revealed the presence of DsRed^+^/CD45^−^ cells in brain, liver and lung. Engraftment occurred in all experimental animals examined. Cells from endometriotic lesions are capable of migration to and engraftment of multiple organs outside of the peritoneal cavity. Micrometastasis of endometriosis is a novel and frequent phenomenon. These data suggest that widespread dissemination of endometriosis may be common, clinically unrecognized and contribute to the diffuse clinical manifestations of this disease.

## INTRODUCTION

Endometriosis is a common gynecological disorder among reproductive aged women (6–10%) [1] due to the deposition and growth of endometrial cells outside the uterus [2, 3]. Approximately, 50% of patients with endometriosis have severe pelvic pain and 40–50% have infertility [4, 5] negatively affecting the health and quality of life of these patients [1, 6]. Even though, the diagnosis of superficial or subtle lesions in endometriosis is still challenging, imaging modalities have increased sensibility and specificity for detecting endometriosis in the abdomen. But the true incidence in the general population is unknown.

Despite being one of the most common gynecologic diseases, the pathogenesis of endometriosis is poorly understood [7, 8]. The most widely accepted theory of its etiology is ectopic implantation, or Sampson’s theory, which proposes that endometrial cells are shed out of the uterus through retrograde menstruation, thereby gaining access to and implanting on pelvic structures [9]. However, implants of endometriosis outside of the peritoneal cavity, in women affected by Mayer-Rokitansky-Kuster-Hauser’s syndrome (absent uterus) or reports of endometriosis in men cannot be explained by these theories [10, 11]. The theory of vascular dissemination suggests that endometrial cells may enter the uterine vasculature or lymphatic system at menstruation and are transported to other sites [12]. Finally, our group has described a stem cell origin of endometriosis; endometriosis arises from ectopic differentiation of circulating mesenchymal stem cells [7, 13–16].

Endometriosis is most commonly found in the pelvis. However, it is also found in organs not contiguous with the peritoneal cavity; these include the pericardium, lung, brain, and skin [3, 7, 8, 17–19]. One review of pathology specimen reports revealed that endometriosis has been identified in nearly all human organs [20]. The exact incidence and pathogenesis of extra pelvic endometriosis is unknown due to the presumed relative rarity of this condition, the lack of effective imaging, and the regression of disease after menopause [17, 21, 22]. It is typically identified at these atypical sites when patients present with atypical, cyclic, clinical symptoms. Women with pleural lesions, in particular, may experience menstrual pneumothorax or hemoptysis [23]. Central nervous system involvement has presented as catamenial headaches, seizures or subarachnoid hemorrhage [17, 24]. These data suggest that dissemination of endometriosis may be widespread.

In this study, we used a murine model of surgically induced endometriosis to determine whether endometriosis-derived cells are capable of migration or micrometastases to distant organs, including the lung, spleen, liver, or brain. Micrometastasis is a form of metastasis where the spread and engraftment of a small number of cells shed from a primary location to another part of the body are too minuscule to be detected by the conventional detection methods [25]. Previously we have identified cell migration from endometriosis within the pelvic cavity to the uterus [26]. We extend those findings and identified the frequent migration of endometriosis-derived cells to extra pelvic organs, suggesting that micrometastasis of endometriosis may be a common phenomenon.

## RESULTS

### Migration of DsRed^+^ cells from experimental endometriosis to lung, spleen, liver, and brain

**Figure 1:**
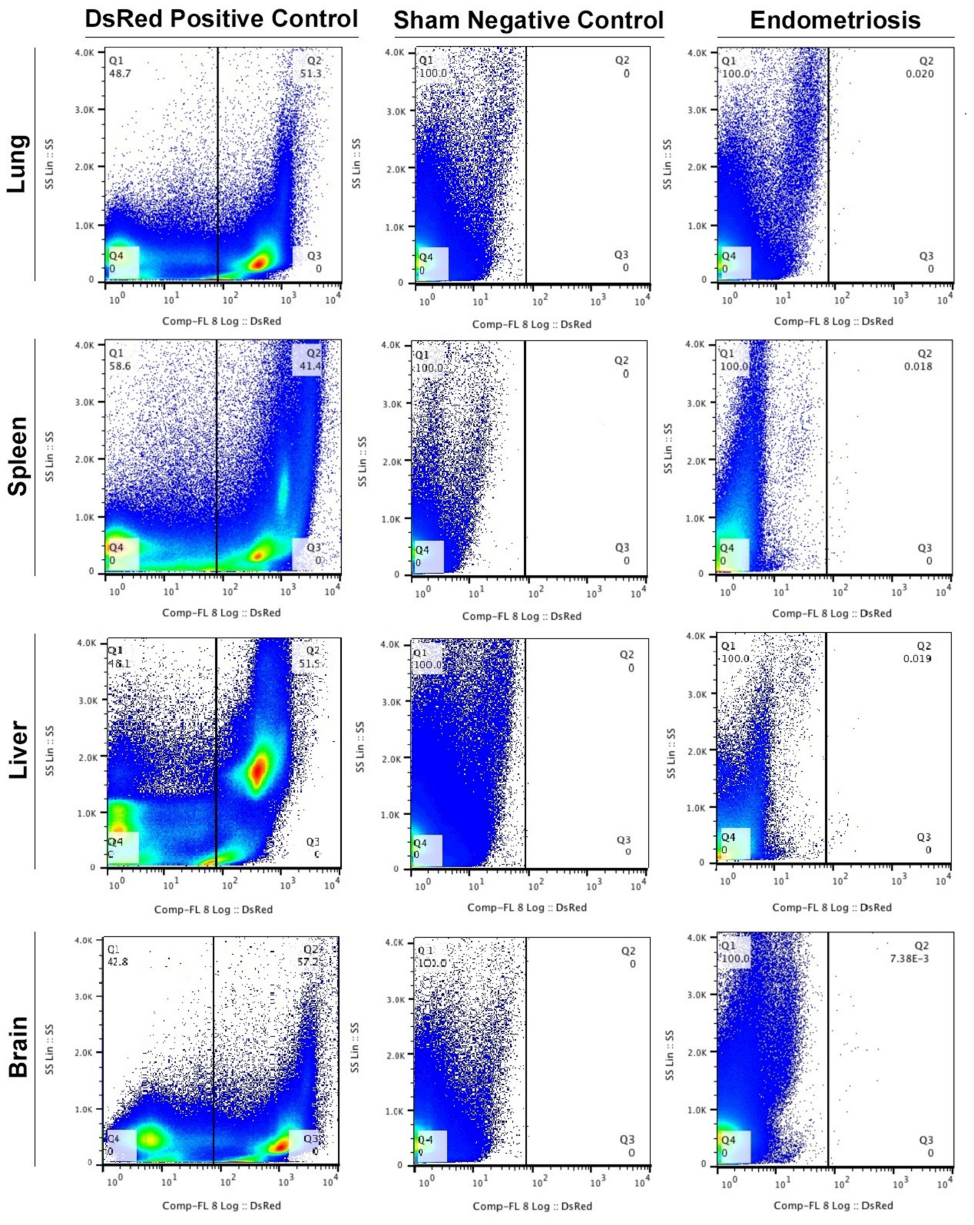
FACS profiles of the DsRed^+^ endometriosis-derived cells in extrapelvic organs Fluorescence Activated Cell Sorting (FACS) was performed to identify DsRed^+^ cells in all animals. (Left to right): Transgenic DsRed mouse (positive control), Sham surgery (negative control) and the experimental endometriosis model. Experiments were performed 3 times with different samples and each time in duplicate. Results (% of cells) presented are the average of triplicates.

**Figure 2:**
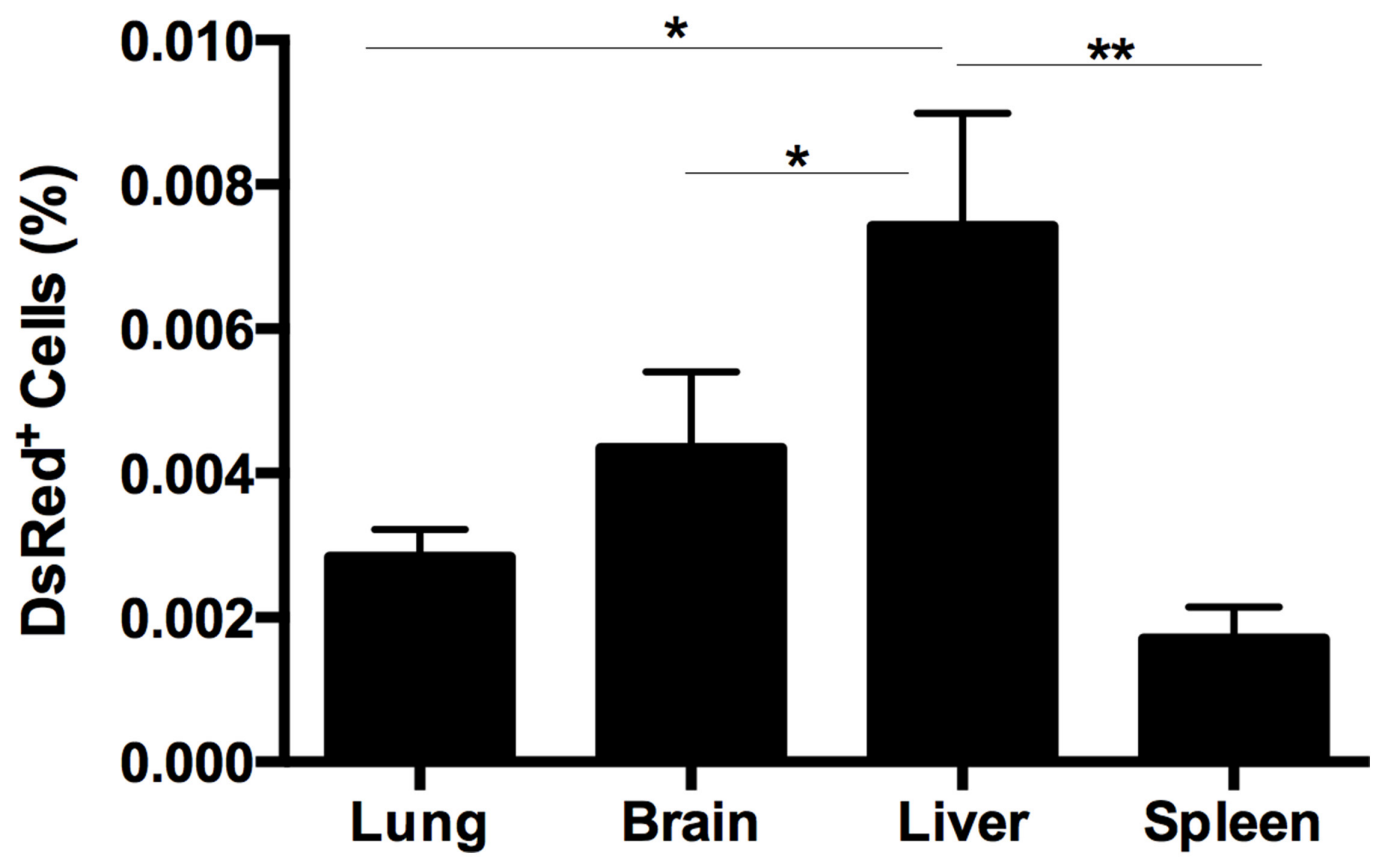
Quantitative analysis of the DsRed expression in lung, spleen, liver, and brain in endometriosis mice FACS analysis was used to quantify the number of DsRed^+^ cells. The number of DsRed, i.e. endometriosis derived, cells was significantly higher in liver compared with lung, brain (*p = 0.01) and spleen (**p = 0.001) in mice eight weeks after induction of endometriosis. Results are expressed as mean ± SEM.

To determine whether endometriosis lesions have cells with the ability to migrate from experimental endometriosis to the lung, we performed Fluorescence Activated Cell Sorting (FACS). As expected, DsRed^+^ cells were found in the lungs of all DsRed^+^ positive control mice. No DsRed^+^ cells were observed in the lungs of the negative controls that underwent sham surgery. Small numbers of DsRed^+^ cells were identified in the lungs of all animals at eight weeks after induction of endometriosis. Experiments were carried out 3 times with different samples and each time in duplicate. (Figure 1). The mean percentage of DsRed^+^ cells in the lungs of animals with endometriosis was 0.0028 ± 0.0003 (Figure 2).

The DsRed^+^ cell population from the spleen in each group (DsRed^+^ positive control, sham negative control and endometriosis model) were evaluated using FACS (Figure 1). An average of 0.0017% ± 0.0004 of the total cells in the spleens of animals with endometriosis were DsRed^+^ (Figure 2). No DsRed^+^ cells were observed in the spleens of the sham controls. Positive controls demonstrated exclusively DsRed^+^ cells (Figure 1). Similarly, analysis of liver cells by FACS revealed an average of 0.0074% ± 0.0015 DsRed^+^ cells in the livers of the mice with experimental endometriosis (Figure 2). Finally, to identify whether endometriosis-derived cells are capable of migration to the brain, the brain cells were subjected FACS as shown in Figure 1. On average 0.0043% ± 0.0010 of the total cells in the brain expressed DsRed^+^ fluorescence in the endometriosis induced mice (Figure 2). The number of DsRed^+^ cells differed in each organ; DsRed^+^ cells were found at the highest frequency in the liver and at the lowest frequency in the spleen. A significantly higher percentage of DsRed^+^ cells was observed in the liver compared to the lung, brain (*p* = 0.01) and spleen (*p* = 0.001) in the endometriosis model (Figure 2).

### Localization of the endometriosis derived cells in each organ

**Figure 3:**
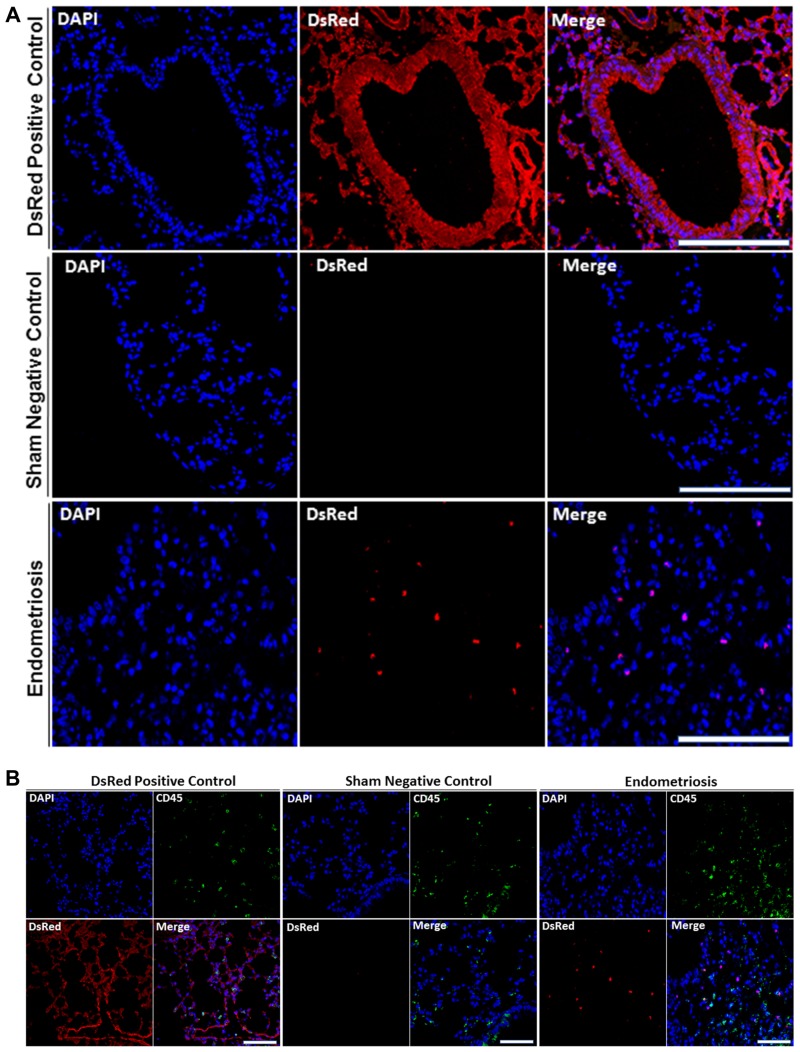
(**A**) DsRed^+^ cells are present in lung parenchyma of mice with endometriosis. Analysis of DsRed^+^ cell localization by confocal microscopy showing intense DsRed immunofluorescence in transgenic DsRed mice. No DsRed expression was observed in sham control mice. In each image, blue represents cell nuclei [4′, 6′- diamino-2-phenylindole (DAPI)], red indicates DsRed. Scale bars, 100 μm (Transgenic DsRed mouse and Sham), 50 μm (Endometriosis). Immunofluorescent images are representative of 3 random fields in each slide, with *n* = 10 mice in each group, in two independent experiments. (**B**) DsRed^+^ cells did not express CD45 in lung parenchyma of mice following induction of endometriosis by using double immunofluorescence. Confocal microscopic view of lung parenchyma. DsRed^+^/Cd45^−^ cells were observed in the lung tissue of mice with experimental endometriosis. CD45 was used as a leukocyte marker to exclude migrating white blood cells. CD45^+^ cells (leukocytes) were observed, as expected, in all models including controls. Blue staining demonstrates cell nuclei [4′, 6′- diamino-2-phenylindole (DAPI)], while red staining corresponds to DsRed, and green staining shows CD45. Scale bars, 100 μm (Transgenic DsRed mouse and Sham), 50 μm (Endometriosis). The negative control tissues have no DsRed cells but have CD45^+^ leukocytes. The positive control DsRed tissues have all DsRed^+^ cells, including the CD45^+^ double positive leukocytes. The experimental mice have DsRed^+^ cells from the endometriosis that are not leukocytes and are therefore CD45^−^. Images are representative of three random fields in each slide, with *n* = 10 mice in each group, in two independent experiments.

**Figure 4:**
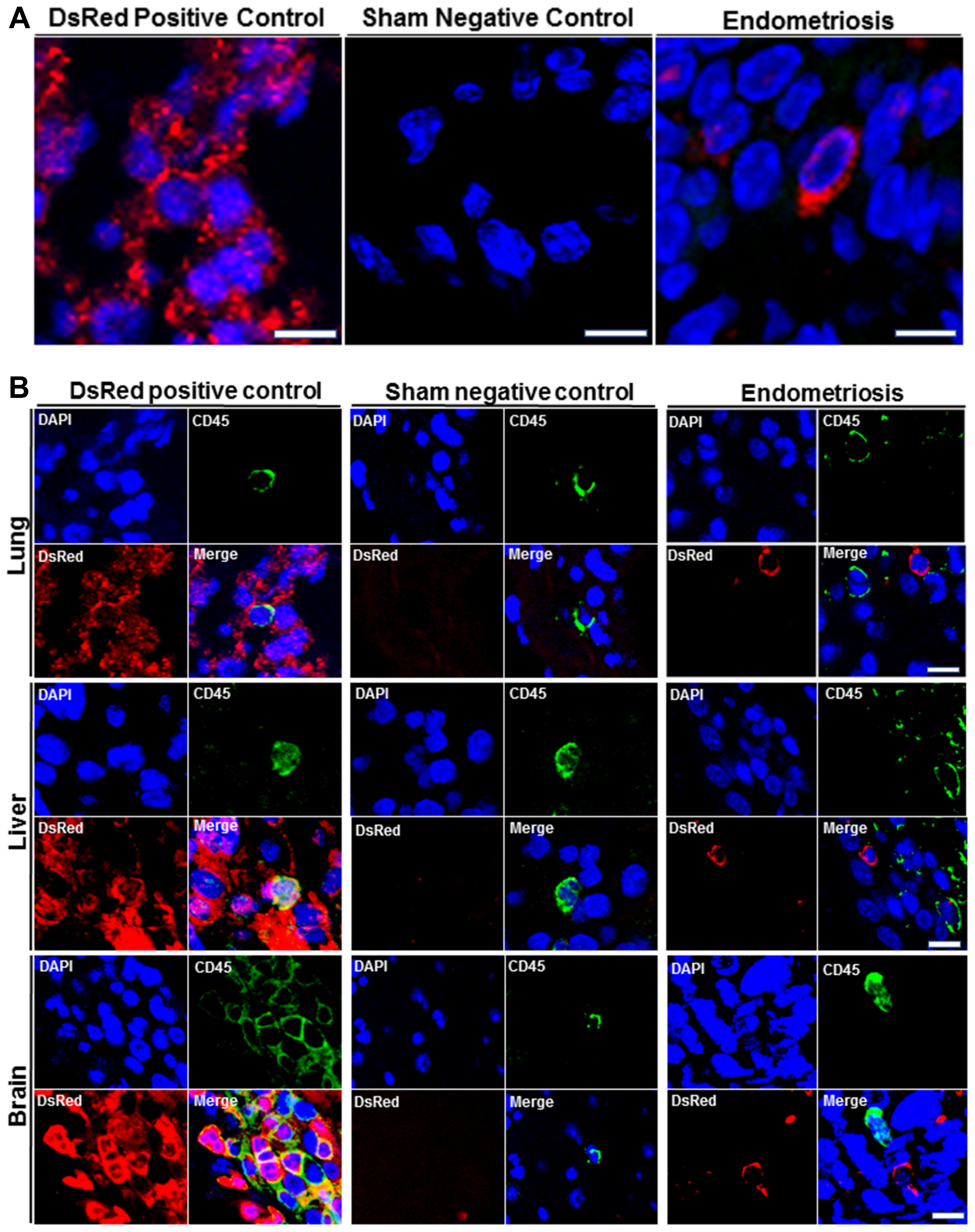
(**A**) High power immunofluorescence confocal images of DsRed expression in lung, liver, and brain. Representative confocal immunofluorescent images were obtained from lung, liver and brain of all animals after staining with DsRed (Red) and nuclei (blue) (left to right): Transgenic DsRed mouse (positive control), Sham (negative control), Experimental endometriosis model. Scale bars, Lung: 5 μm (Transgenic DsRed mouse and Endometriosis), 10 μm (Sham); Liver: 2.5 μm (Transgenic DsRed mouse and Endometriosis), 5 μm (Sham); Brain: 4 μm (Transgenic DsRed mouse and Endometriosis), 8 μm (Sham). Images are representative of three random fields in each slide, with *n* = 10 mice in each group, in two independent experiments. (**B**) Double immunofluorescence (DsRed/CD45) analysis of extrapelvic organs. Analysis by immunofluorescence of lung, liver and brain sections from all animals. DsRed^+^ cells did not express CD45 in the extrapelvic organs of mice following induction of endometriosis. CD45 was used as a leukocyte marker to exclude migrating white blood cells. DsRed^–^/CD45^+^ cells (leukocytes) were observed in transgenic DsRed and sham model mice. Blue staining demonstrates cell nuclei [4′, 6′- diamino-2-phenylindole (DAPI)], while red staining corresponds to DsRed, and green staining shows CD45 positivity. Scale bars, Lung: 5μm (Transgenic DsRed mouse and Endometriosis), 10 μm (Sham); Liver: 5 μm, Brain: 10 μm. Images are representative of 3 random fields in each slide, with *n* = 10 mice in each group, in two independent experiments.

We then sought to confirm the presence of DsRed^+^ cells migrating from endometriosis to the multiple extra pelvic organs. Therefore, we examined paraffin-embedded tissue sections using immunostaining. Lung, liver and brain sections of all animals were visualized under a fluorescence microscope in two independent experiments. Figure 3A displays representative confocal micrographs of lung parenchyma from our three different groups consisting of the DsRed (positive control), sham surgery (negative control) and endometriosis-induced mice. Lung tissue from DsRed^+^ mice was highly fluorescent and demonstrated exclusively DsRed expressing cells as shown in Figure 3A. Mice that had undergone sham surgery displayed no DsRed fluorescence and DsRed expressing cells were observed in the lung tissue of mice with experimental endometriosis. Moreover, DsRed^+^ cells were most frequently observed in the bronchial compartment of lungs of endometriosis mice. CD45, a pan leukocyte marker, was used to identify leukocytes within the lesions. The DsRed^+^ cells identified in the lungs of mice with endometriosis did not express CD45. This suggests that the migratory cells were not leukocytes. As expected, no DsRed^+^ cells were observed in the sham model, while CD45^+^ cells were observed (Figure 3B). High power view of the lung tissue demonstrating CD45^−^/DsRed^+^ cells in the lung are shown in Figure 4A and 4B, respectively. In the same way, we examined whether endometriosis-derived cells were capable of migration to the liver in the mice with endometriosis. After immunofluorescent labeling, DsRed^+^ cells were found to localize in the liver parenchyma (Figure 4A). We found no DsRed^+^ cells in sham model livers. Then, we performed double staining for DsRed and CD45 on all three groups. The DsRed^+^ cells did not express CD45 in the endometriosis mice. These findings were similar to those of the other organs, indicating the nonhematopoietic origin of the endometriosis-derived DsRed^+^ cells (Figure 4B). Finally, the presence of DsRed^+^ cells in the cerebral tissue of the endometriosis model mice was analyzed using immunofluorescent staining. No DsRed expression was observed in sham model mice, whereas the DsRed positive control tissue sections demonstrated expected strong DsRed positivity (Figure 4A). Notably, in the recipient mice, the migrated DsRed^+^ cells in the brain appeared predominantly in the hypothalamus, hippocampus, and superficial layer of the cerebral cortex. DsRed^+^ cells were negative for CD45 in the mice with experimental endometriosis (Figure 4B). These findings again support the nonhematopoietic origin of the endometriosis-derived DsRed^+^ cells.

## DISCUSSION

We demonstrate here that endometriosis-derived cells are capable of migration to extrapelvic organs including the lung, spleen, liver and brain. The presence of endometriosis-derived cells in all of the endometriosis mice tested suggests that migration of endometriosis to organs outside of the pelvis is common. Microscopic implants of endometriosis may be biologically functional and may explain some of the systemic manifestations of endometriosis in humans. As endometriosis is associated with various systemic abnormalities in humans, the relatively high recruitment of cells from the transplanted endometrial tissue to the brain and liver is particularly interesting.

Endometriosis is generally considered to be a reproductive tract disease, causing pelvic pain and infertility [27]. However women with endometriosis often experience a more broad spectrum of debilitating symptoms including non-pelvic pain, fatigue, malaise, eating disorders, anthropometric variation, endocrine and metabolic dysfunction, immunologic defects, and sociopsychological issues that impair their quality of life [28–33]. The underlying mechanisms that produce this wide array of symptoms that transcend the pelvis are unknown [34]. We speculate that some may be due to undiagnosed cellular infiltration with endometriosis. The most common sites of endometriosis in humans are the ovaries, anterior and posterior uterine culs-de-sac, posterior broad ligaments, uterosacral ligaments, uterus, fallopian tubes, sigmoid colon, appendix, and uterine round ligaments [35]. While extrapelvic endometriosis is thought to be rare, it has been found in the gastrointestinal tract (32.3%), urinary tract (5.9%), and distant sites including the lung, umbilicus, skin scars, liver, gallbladder, pancreas, breast, and extremities [36, 37]. Involvement of the vertebrae, bone, peripheral nerves, diaphragm, spleen, and central nervous system have also been reported [38]. Here, we describe the common and uniform occurrence of micrometastasis of endometriosis to multiple organs.

We demonstrated the presence of endometriosis-derived cells in the bronchial compartment in a murine model. Thoracic endometriosis is the most common extra-pelvic location in humans and primates [37, 39]. Thoracic endometriosis can involve the pleura, parenchyma, diaphragm, and bronchi, which may present as catamenial pneumothorax, hemothorax, hemoptysis, chest pain, or dyspnea [39, 40]. Intrathoracic endometriosis lesions probably go unrecognized due to inaccessibility of these lesions at imaging or bronchoscopy [39, 41]. More subtle variants may exist that result in symptoms that are ignored or dismissed.

Clinically, endometriosis within the liver has been reported; however, it is rarely recognized [42, 43]. Hepatic endometriosis has resulted in jaundice and portal vein thrombosis [43]. Unusual symptoms such as refractory right shoulder pain have additionally been a presenting symptom [44]. Hepatic endometriosis has appeared as solitary nodules or as multiple intra/extra hepatic cystic lesions on imaging [45]. We observed a relatively high recruitment of cells from the transplanted endometrial tissue to the liver, which is particularly interesting as endometriosis is associated with various metabolic abnormalities, including low BMI and metabolic hormone dysregulation [28, 46]. Hepatic involvement may be a precursor of metabolic alterations. The presence of micrometastasis to the liver may help in understanding some of the various systemic metabolic manifestations in endometriosis. Involvement of endometriosis in the Central Nervous System (CNS) has presented as catamenial headache, seizure, papilledema, subarachnoid hemorrhage, and gait disturbance [17, 47]. Women with endometriosis are more likely to suffer from migraines [48]. There is no classic imaging finding that leads to the diagnosis of endometriosis in the CNS making the true incidence impossible to discern. Similarly, the hypothalamus is the “biological clock” of the neuroendocrine system. It influences appetite, sleep and pituitary gland action, among other functions [49, 50]. The hypothalamus-pituitary axis has been reported to be compromised in endometriosis, however the underlying mechanisms are unclear [51]. An association between endometriosis and mood disorders has also been previously demonstrated [52]. Women with endometriosis have a higher risk of depression and anxiety than control subjects [53]. Finally, dysregulation of pain perception is also believed to be common in endometriosis [54]. Perhaps some of these clinical manifestations may be due to undetected endometriosis in the CNS. During the short time course of these experiments we did not detect any physiologic manifestations. In humans we suspect that the much longer duration of accumulation may lead to clinical manifestations. Endometriosis has many related systemic symptoms that are currently poorly explained. Some may be due to clinically unrecognized endometriosis micrometastasis.

In Summary, we observed that endometrial-derived cells migrated to extrapelvic organs. Our findings suggest that endometriosis in locations distant from the pelvis may be more common than previously recognized. Endometriosis should be considered a systemic disease that is often subclinical. Single cells or small implants of endometriosis likely may go unrecognized clinically. Our findings may explain the diffuse symptoms that are often considered components of endometriosis. Migration of endometriosis-derived cells to distant organs suggests a contribution of these cells to a wide range of previously unexplained clinical manifestations, including refractory pain, appetite and eating behaviors, pain perception, altered metabolism, and immune dysfunction. Blocking of cellular migration from ectopic lesions to distant areas may be a therapeutic target. Further investigation is necessary to characterize these migratory cells and the potential mechanism of endometriosis-derived cells metastasis in endometriosis.

## MATERIALS AND METHODS

### Animal care

Eight weeks old female transgenic DsRed (Discosoma sp. Red Fluorescent Protein) mice (20) from Jackson Laboratories (Bar Harbor, ME, USA) and female C57BL/6 mice (20) were kept under controlled conditions (a 12-hour light, 12-hour dark cycle, at 22°C) with free access to water and chew. Animals were treated in accordance with a protocol approved by the Yale University Institutional Animal Care and Use Committee (IACUC), and the U.S. Principles for Utilization and Care of Vertebrate Animals Used in Testing, Research, and Training were followed. Briefly, the female transgenic DsRed mice were collected from litters of the same age from different dams of C57BL/6 background. Both parents have construct containing a Red Fluorescent Protein gene (DsRed.MST) under the control of a chicken beta actin promoter.

### Murine experimental model of endometriosis

Twenty C57BL/6 mice were randomly assigned to one of two groups, endometriosis group (*n* = 10) or sham control group (*n* = 10). Experimental endometriosis was created in ten female C57BL/6 mice with intact ovaries as previously described [55]. Briefly, two pieces (7 millimeters) of uterine horn were obtained from transgenic DsRed donor mice. The tissue pieces were split longitudinally to expose the lumen and implanted into the pelvic peritoneum of recipients, bilaterally. Simultaneously, female C57BL/6 mice underwent sham surgery by leaving only suture material in a similar location. Matched DsRed mice were used as a positive control (*n* = 10).

### Tissue collection and assessment

Eight weeks after creation of experimental endometriosis, animals were sacrificed and multiple organs including lung, spleen, liver, and brain were harvested. Fresh tissues were assessed by Fluorescence Activated Cell Sorting (FACS). The remaining tissue was stored in 4% paraformaldehyde overnight, transferred to 70% ethanol, and then embedded in paraffin for immunofluorescent analysis.

### Fluorescence-activated cell sorting (FACS)

Lung, spleen, liver, and brain tissue were assessed to determine the presence of DsRed^+^cells using FACS. The specimens were finely minced with surgical scalpels, and subsequently digested with a solution of Hank’s Balanced Salt Solution (Life Technologies, Inc, Invitrogen, Carlsbad, CA, USA) containing HEPES (25 mM), collagenase B (1 mg/ml, Roche Diagnostics, Indianapolis, IN, USA), and deoxyribonuclease (0.1 mg/ml, Sigma-Aldrich, St. Louis, MO, USA) for 60–90 min at 37°C. All samples were filtered using 70-μm cell strainers and centrifuged at 3,000 rpm at 4°C for 5 minutes. Cell pellets were suspended in FACS buffer and then sorted via fluorescence activated cell sorting (FACS) on a Beckman Coulter MoFlo machine (Beckman Coulter, San Jose, CA, USA). Acquired data were analyzed using the FlowJo V10 software (Tree Star, Ashland, OR, USA) for three independent experiments.

### Paraffin-embedded tissue immunostaining

Paraformaldehyde-fixed specimens were paraffin embedded, cut into 3-μm sections, mounted on glass slides, and stained for DsRed and CD45, a hematopoietic cell marker mainly found on leukocytes. Six sections (one/slide) were prepared from each organ of interest of every animal. Three random regions from each organ of the lung, liver and brain were selected in two independent experiments. Sections were deparaffinized and rehydrated through a series of 15-minute xylene and 25-minute ethanol washes and rinsed in Phosphate-Buffered Saline (PBS). The slides with sections were then placed in boiling sodium nitrate (pH = 6) for 10 min and then treated for 15 min with ammonium chloride in PBS to protect against auto-fluorescence. Sections were incubated in blocking buffer using 5% donkey serum (Invitrogen) for 30 min at room temperature followed by primary antibodies: polyclonal goat anti-DsRed IgG antibody (Santa Cruz Biotechnology, Dallas, TX, USA), and rat anti-CD45 antibody (Abcam, Cambridge, MA, USA) diluted 1/200 in 5% donkey serum and kept in a humidified chamber overnight at 4°C. Then, the sections were rinsed three times for 20 min respectively in PBS on an orbital shaker and incubated with the following secondary antibodies: donkey anti-goat Alexa Fluor 568 (Life Technologies, Grand Island, NY, USA), and donkey anti-rabbit Alexa Fluor 488 (Life Technologies, Grand Island, NY, USA) diluted 1/500 in PBS for one hr, away from light, at room temperature. Sections were mounted with Vectashield mounting medium with DAPI (4´6-diamidino-2-phenylindole, Vector Laboratories, Burlingame, CA, USA) for 15 min and immunofluorescence signals were observed using a laser scanning confocal microscope (LSM 710, Zeiss, New York, NY, USA). A minimum of 100 high-power microscopic fields were analyzed from triplicate slides in each sample, in three independent experiments. The investigators who performed the tissue investigations were blinded to the experimental groups.

### Statistical analysis

Data were analyzed using GraphPad Prism 5 software (Graph-Pad Software, San Diego, CA, USA) and are presented as the Mean ± SEM from at least three independent experiments. One-sided analysis of variance (ANOVA) was used to analyze differences in multiple organs comparisons, followed by Tukey’s post hoc test. Values were considered to be statistically significantly different if *p* < 0.05.
